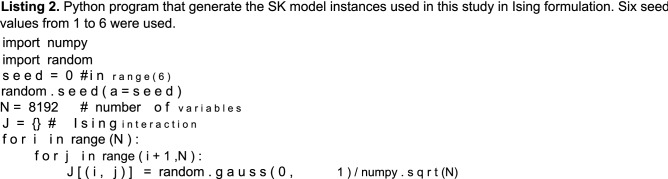# Benchmark of quantum-inspired heuristic solvers for quadratic unconstrained binary optimization

**DOI:** 10.1038/s41598-022-06070-5

**Published:** 2022-02-09

**Authors:** Hiroki Oshiyama, Masayuki Ohzeki

**Affiliations:** 1 Graduate School of Information Sciences, Tohoku University, Sendai, 980-8579 Japan; 2 Institute of Innovative Research, Tokyo Institute of Technology, Yokohama, 226-8503 Japan; 3 Sigma-I Co., Ltd., Tokyo, 108-0075 Japan

**Keywords:** Applied physics, Information theory and computation, Quantum physics

## Abstract

Recently, inspired by quantum annealing, many solvers specialized for unconstrained binary quadratic programming problems have been developed. For further improvement and application of these solvers, it is important to clarify the differences in their performance for various types of problems. In this study, the performance of four quadratic unconstrained binary optimization problem solvers, namely D-Wave Hybrid Solver Service (HSS), Toshiba Simulated Bifurcation Machine (SBM), Fujitsu Digital Annealer (DA), and simulated annealing on a personal computer, was benchmarked. The problems used for benchmarking were instances of real problems in MQLib, instances of the SAT-UNSAT phase transition point of random not-all-equal 3-SAT (NAE 3-SAT), and the Ising spin glass Sherrington-Kirkpatrick (SK) model. Concerning MQLib instances, the HSS performance ranked first; for NAE 3-SAT, DA performance ranked first; and regarding the SK model, SBM performance ranked first. These results may help understand the strengths and weaknesses of these solvers.

## Introduction

Quantum annealing (QA)^1,2^, which is a quantum heuristic algorithm for solving combinatorial optimization problems, has attracted a great deal of attention because it is implemented using real quantum systems by D-Wave Systems Inc.^3,4^, aiming at becoming more powerful than classical algorithms such as simulated annealing (SA)^5,6^. To use the current D-Wave’s QA device, a combinatorial optimization problem must be mapped to a quadratic unconstrained binary optimization (QUBO) problem. QUBO is an optimization problem of binary variables ${\mathrm{x}}_{{\mathrm{i}}} \in \left\{ {0, \, 1} \right\}$, where ${\mathrm{i}} \in \left\{ {{1},{ 2}, \ldots ,{\mathrm{ N}}} \right\}$, and its cost function to be minimized is defined as

1 $${\mathrm{E}}({\mathbf{x}}) = \sum\limits_{{\mathrm{i,j}}} {{\mathrm{Q}}_{{\mathrm{i,j}}}\, {\mathrm{x}}_{{\mathrm{i}}}\, {\mathrm{x}}_{{\mathrm{j}}} } ,$$

where Q_i, j_ is a real number called QUBO matrix element. In general, QUBO is NP-hard^7^, and many NP-complete problems and combinatorial optimization problems are mapped to QUBO^8^.

Although current QA devices have limited capability owing to hardware implementation limitations, in anticipation of future developments of QA devices, methods using QUBO models for solving real-world problems in a variety of fields have been actively studied^9–15^. Inspired by this trend, several sophisticated heuristic QUBO solvers have been developed and commercialized^16–19^. It is highly non-trivial to determine whether a particular algorithm is more powerful than another because the performance of heuristic algorithms varies depending on the target problem. For successful application to real-world problems and further development of these QUBO solvers, it is necessary to clarify the strengths and weaknesses of each solver for various types of QUBO problems. In this study, we benchmarked the performance of three commercialized QUBO solvers including one using a real QA device: D-Wave Hybrid Solver Service (HSS), Toshiba Simulated Bifurcation Machine (SBM), and Fujitsu Digital Annealer (DA). In order to understand the characteristics of the solvers, we benchmark various types of problems, including Ising spin glass problems and real-world problems. This is in contrast to a similar benchmark study reported recently^20^, which used only a single kind of constraint satisfaction problem (specifically, 3-regular 3-XORSAT). While in Ref.^20^, the size dependence of the time to obtain an optimal solution with a certain probability is analyzed in detail, in this study, the performance of the solvers is evaluated by comparing the value of the cost function obtained for a given execution time. Such a performance evaluation will be helpful in application cases where approximate solutions are acceptable.

The remainder of this paper is organized as follows. In “QUBO solvers” section, we briefly explain the solvers benchmarked. In “Problem instances for benchmarking” section, the definition of the problem instances used for benchmarking are provided. In “Results” section, we present the results of the benchmarking experiment. Concluding remarks are given in “Discussion and conclusion” section.

## QUBO solvers

In this section, we briefly explain the four solvers used in this study. Three commercial solvers were benchmarked. For comparison, we also experimented with SA on a personal computer.

The first solver is HSS, commercialized by D-Wave Systems Inc.^16^. This solver is a so-called quantum–classical hybrid algorithm that employs QA as an accelerator. Note that the actual implementation of the algorithm is not open to the public. Thus, it is unclear how QA is used internally. We used HSS hybrid BQM solver, version 2.0, which can manage up to 10^6^ variables and 2 × 10^8^ couplings^21^. We accessed HSS via Leap cloud.

The second solver is SBM, commercialized by Toshiba^18^. The QA inspired algorithm of SBM, so-called simulated bifurcation (SB) algorithm, uses the adiabatic time evolution of Kerr-nonlinear parametric oscillators (KPOs)^22^. The dynamics in the classical limit of KPOs can be quickly computed in classical computers by solving the independent equations of motion in parallel^18^. To overcome accuracy degradation caused by analog errors due to the use of the dynamics of continuous variables, a variant of the SB algorithm called ballistic SB (bSB) algorithm was developed, which mitigate the analog error by modifying the potential term of the equation of motion. As a further improvement of the bSB algorithm, the discrete SB (dSB) algorithm was also developed, which reduces the analog error by discretizing the potential term of the bSB algorithm^23^. We use SBM evaluation version 1.5.1 (which is not publicly available), that uses dSB algorithm and can manage all-to-all coupling of up to 10^6^ variables and 10^8^ nonzero couplings. Parallelization is 80 or 160 per GPU. In this study, we used the autoising solver; hyperparameters are automatically searched by the solver. We accessed SBM via evaluation version directly from Toshiba.

The third solver is DA, commercialized by Fujitsu^19^. DA uses an SA-specific hardware architecture to accelerate the parallel tempering Markov chain Monte Carlo (MCMC) calculation^24,25^. Although DA does not use quantum algorithms, it is inspired by D-Wave devices in the sense that the hardware is specialized for QUBO solving. We used fujitsuDA2PT solver, which can manage all-to-all coupling of up to 8192 variables. We accessed DA via DA Center Japan.

For comparison with these commercial solvers, we ran SA using the open-source software D-Wave neal, version 0.5.7^26^, on a personal computer with Ubuntu 20.04.3 LTS and Python 3.8.2. D-Wave neal implements SA with MCMC without parallel tempering method. The CPU used in the experiment was Intel Core i9-9900K, and single-threaded runs were performed.

## Problem instances for benchmarking

In this section, we explain the three problem sets used in the conducted benchmarking.

### MQLib repository instances

We used the same set of 45 problems used in the benchmarks presented in HHS’s white paper^16,27^. This problem set is extracted from the MQLib repository, and some of the problems have their origin in real-world problems, such as image segmentation^28^. This problem set was reported to be time-consuming to solve because of all the heuristics contained in the MQLib library. Concerning benchmarking, a 20-minute run is recommended for each problem^16^. The 45 problems are uniformly classified into nine classes: three classes according to size (small: 1000 ≤ N ≤ 2500, medium: 2500 < N ≤ 5000, and large: 5000 < N ≤ 10000) and three classes according to edge density (sparse: d ≤ 0.1, medium: 0.1 < d ≤ 0.5, and dense: 0.5 < d), where d is the number of edges divided by the number of edges in a complete graph of the same size^16^.

### Not-All-Equal 3-SAT

Satisfiability problem (SAT) is one of the most fundamental NP-hard problems and therefore it is good benchmark problem for heuristic solvers. Not-all-equal 3-SAT (NAE 3-SAT) is a variant of the Boolean SAT problem and is an NP-complete problem^29^. NAE 3-SAT requires at least one literal to be true and at least one literal to be false in each clause with three literals. The cost function of a random NAE 3-SAT with N variables and M clauses is expressed in a straightforward manner in the Ising model with σ_i_ ∈ {− 1, 1}, where 1 ≤ i ≤ N:

2 $${\mathrm{E}}({{\varvec{\upsigma}}}) = \frac{1}{4}\sum\limits_{{{\mathrm{m}} = 1}}^{{\mathrm{M}}} {\left( {{\varsigma }_{{{\mathrm{m}},1}} {\varsigma }_{{{\mathrm{m}},2}}\upsigma _{{\mathrm{im,1}}}\upsigma _{{\mathrm{im,2}}} + {\varsigma }_{{\mathrm{m,2}}} {\varsigma }_{{\mathrm{m,3}}}\upsigma _{{\mathrm{im,2}}}\upsigma _{{\mathrm{im,3}}} + {\varsigma }_{{\mathrm{m,3}}} {\varsigma }_{{\mathrm{m,1}}}\upsigma _{{\mathrm{im,3}}}\upsigma _{{\mathrm{im,1}}} + 1} \right)} ,$$

where i_m,l_ ∈ {1, 2, … ,N} and ζ_m,l_ ∈ {− 1, 1} for 1 ≤ m ≤ M and 1 ≤ l ≤ 3 are random variables that follow a discrete uniform distribution; ζ_m,l_ =  − 1 corresponds to the negation of the l-th Boolean variable in clause m. Each clause has three different variables, i.e., i_m,l_ / = i_m,l_′ if l / = l′. If the minimum of E(**σ**) in Eq. (2) is 0 for a given formula, it is satisfiable (SAT); otherwise, it is unsatisfiable (UNSAT). The QUBO formulation as in Eq. (1) can be easily obtained from this Ising formulation by the variable transformation x_i_ = (σ_i_ + 1)/2. Because NAE 3-SAT has such a natural QUBO representation, it is a suitable benchmark problem for QUBO solvers amongst SAT variants. When the clause-to-variable ratio is M/N = 2.11, the SAT-UNSAT phase transition occurs, and problem instances are most difficult to solve^30–32^. In this study, we used randomly generated instances with this critical clause-to-variable ratio for benchmarking.

### Sherrington–Kirkpatrick model

The Sherrington–Kirkpatrick (SK) model is an Ising spin glass model with infinite spatial dimensions^33,34^. The cost function of N variables with no external field is expressed as

3 $${\mathrm{E}}({{\varvec{\upsigma}}}) = \frac{1}{{\sqrt {\mathrm{N}} }}\sum\limits_{{1 \le {\mathrm{i}} \le {\mathrm{j}} \le {\mathrm{N}}}} {{\mathrm{J}}_{{\mathrm{i,j}}}\upsigma _{{\mathrm{i}}}\upsigma _{{\mathrm{j}}} } ,$$

where J_i, j_ is a random Gaussian variable. As previously explained, the QUBO formulation can be easily obtained. The mean field analysis shows that the energy landscape of the SK model has a many-valley structure separated by asymptotically infinitely large energy barriers, which implies that it is extremely difficult to find the exact solution^35^. In this study, we used randomly generated instances with J_i, j_ presenting zero mean and unity standard deviation for benchmarking.

## Results

In this section, we present benchmarking results for each of the three problem sets introduced in the previous section. In the results shown below, the network time required to send the instance and receive the result was ignored in the measurement of execution time. Regarding HSS, the number of seconds specified in time_limit was used as the execution time. For SBM, the time specified in timeout was used as the execution time. Concerning DA, there was no parameter to specify the execution time directly, so total_elapsed_time recorded in the response file was used as the execution time. Finally, for SA with D-Wave neal, we measured the time taken for the sample function to finish.

### MQLib instances

First, we present the results for a 5-min experiment of the instances from the MQLib repository. For HSS and SBM, the execution time was set to 5 min. For DA, number_replicas was set to 128 and number_iterations was adjusted for each instance so that the deviation of execution time in 5 min was within 20 s. Concerning SA, num_sweeps was adjusted for each instance such that the execution time was 5 min.

**Figure 1:**
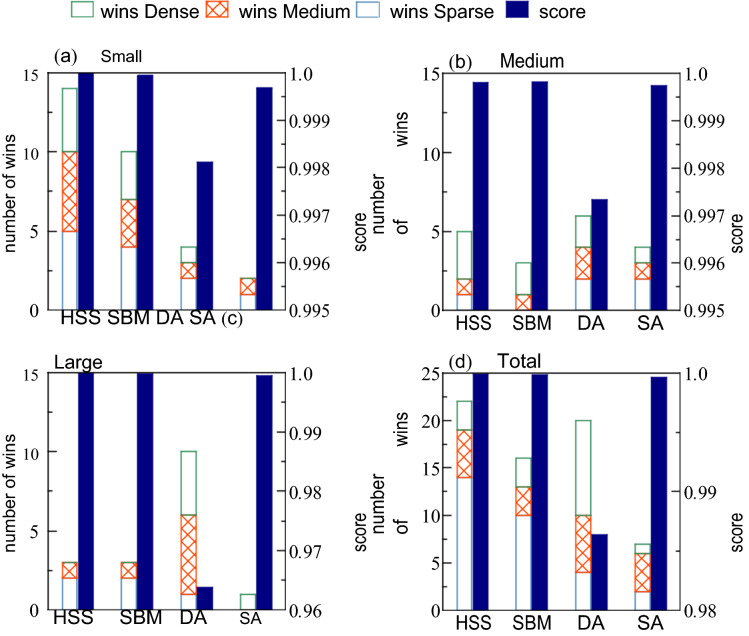
Number of wins (left axis) and average score (right axis) for a 5-min experiment of MQLib instances. Each panel shows the result for a class categorized by problem size, (**a**) Small, (**b**) Mediun, and (**c**) Large; (**d**) Total number of wins and average score for all instances. The score for each instance is defined by Eq. (4). In calculating the average score, instance g000644 was ignored due to absence of data for DA.

Figure 1 shows the number of wins for each solver; this number was counted when the solver obtained the best solution. If there was more than one solver with the best solution, the number of wins was counted for all of them. The total result for all classes was that HHS won most of the problems (22), followed by DA (20), SBM (16), and SA (7). The results for each class classified by size show that HSS won the most for the small class, while DA won the most for the medium and large classes. The results for each class classified by edge density show that, for Sparse class, HSS won the most, for Medium class, DA won the most, and HSS and DA won the most. The number of wins of SA was only 2 at most, and most of the time, it was 0 or 1 for each of the nine classes.

Furthermore, we evaluate the quality of the obtained solution using a score defined as the ratio of the value of cost function

4 $$\begin{aligned} & ({\mathrm{E}}_{{{\mathrm{solver}}}} = \, \{ {\mathrm{E}}_{{{\mathrm{HSS}}}} ,{\mathrm{ E}}_{{{\mathrm{SBM}}}} ,{\mathrm{ E}}_{{{\mathrm{DA}}}} ,{\mathrm{ E}}_{{{\mathrm{SA}}}} \} ){\mathrm{ to}}\,{\mathrm{the}}\,{\mathrm{best}}\,{\mathrm{value}}\,{\mathrm{obtained}}\,{\mathrm{in}}\,{\mathrm{this}}\,{\mathrm{experiment}} \\ & \quad \quad ({\mathrm{E}}_{0} = {\mathrm{ min}}\{ {\mathrm{E}}_{{{\mathrm{HSS}}}} ,{\mathrm{ E}}_{{{\mathrm{SBM}}}} ,{\mathrm{ E}}_{{{\mathrm{DA}}}} ,{\mathrm{ E}}_{{{\mathrm{SA}}}} \} ){:} \\ & \quad {\mathrm{S}}_{{{\mathrm{solver}}}} = {\mathrm{ E}}_{{{\mathrm{solver}}}} /{\mathrm{E}}_{0} ({\mathrm{solver}} \in \{ {\mathrm{HSS}},{\mathrm{ SBM}},{\mathrm{ DA }},{\mathrm{ SA}}\} ). \\ \end{aligned}$$

Tables 1, 2, 3, 4, 5, 6, 7, 8 and 9 show the score for each instance, and Fig. 1 shows the average of the scores for Small, Medium, and Large classes, and for all instances. The original lowest values of the cost function found in this benchmarking are listed in Table 10. The average scores of HSS and SBM are almost identical and higher than other solvers. This implies that HSS and SBM have stable performance on a wide range of problems. On the other hand, DA has an exceptionally bad solution for the instance g001345, which is why the average score drops significantly in the Large class. In addition, in the Small and Medium classes, the average score of DA is about 0.01 lower than the other solvers. This implies that DA is slightly less stable, because even for SA, which has the fewest wins, the difference in average score from HSS is within 0.001.

**Table 1:** Values of score, defined by Eq. (4) for small and sparse classes.

Input	Size	Density	HSS	SBM	DA	SA
g000989	2319	0.00086	**1.0**	**1.0**	**1.0**	0.998708
g003215	2206	0.00093	**1.0**	0.999457	0.998103	0.997985
g001269	2294	0.0017	**1.0**	**1.0**	**1.0**	0.999847
g000421	2034	0.0038	**1.0**	**1.0**	0.985010	0.999303
g002440	2242	0.044	**1.0**	**1.0**	0.999213	**1.0**

The first row shows the instance name, the second row presents the number of variables, the third row contains the edge density, and the fourth and subsequent rows show the results for each solver. The values are computed in single precision from the obtained solution of binary variables; they are shown with six decimal places. The best solutions obtained in this benchmarking are shown in bold.

**Table 2:** Results for small and medium classes, same as Table 1.

Input	Size	Density	HSS	SBM	DA	SA
g000432	2153	0.11	1.0	0.999958	0.999045	0.999974
g000524	2218	0.14	1.0	1.0	1.0	1.0
g002586	2079	0.16	1.0	1.0	0.999102	0.999890
g001327	2318	0.3	1.0	1.0	0.999300	0.999928
g001469	2412	0.46	1.0	0.999824	0.998105	0.999911

**Table 3:** Results for small and dense classes, same as Table 1.

Input	Size	Density	HSS	SBM	DA	SA
g002600	2432	0.85	1.0	0.999999	0.999505	0.999976
g000969	2453	0.86	1.0	1.0	0.995138	0.999645
g002898	2041	0.86	1.0	1.0	1.0	0.999996
g001581	2383	0.86	0.999999	1.0	0.999640	1.000000
g000788	2342	0.88	1.0	0.999860	0.999492	0.999959

**Table 4:** Results for medium and sparse classes, same as Table 1.

Input	Size	Density	HSS	SBM	DA	SA
g000377	3398	0.00069	0.998763	0.999890	1.0	0.998353
g002569	2815	0.0011	1.0	0.999459	0.983877	0.998564
g001086	3706	0.0016	0.998913	0.998673	0.985686	1.0
g001337	2850	0.051	0.999975	0.999923	1.0	0.999931
g000283	3364	0.072	0.999946	0.999905	0.997073	1.0

**Table 5:** Results for medium and medium classes, same as Table 1.

Input	Size	Density	HSS	SBM	DA	SA
g002512	4731	0.12	0.999913	0.999861	1.0	0.999980
g000802	3956	0.13	0.999990	1.0	0.998449	0.999919
g003059	3447	0.14	0.999973	0.999939	1.0	0.999962
g002332	3181	0.22	0.999994	0.999996	0.999156	1.0
g002034	2528	0.35	1.0	0.999997	0.999201	0.999979

**Table 6:** Results for medium and dense classes, same as Table 1.

Input	Size	Density	HSS	SBM	DA	SA
g003198	3972	0.74	1.0	0.999956	0.999616	0.999979
g002207	2677	0.74	1.0	1.0	1.0	0.999954
g001913	3865	0.75	1.0	0.999786	0.999333	0.999643
g001393	3938	0.83	0.999967	1.0	1.0	0.999886
g002370	3884	0.84	0.999716	0.999843	0.997744	1.0

**Table 7:** Results for large and sparse classes, same as Table 1.

Input	Size	Density	HSS	SBM	DA	SA
imgseg-216041	7724	0.00039	1.0	0.999919	0.996163	0.995890
imgseg-376020	7455	0.00049	1.0	0.999522	0.989190	0.998811
g001883	6831	0.00059	1.000000	1.0	0.999489	0.999998
g000644	10,000	0.0016	0.999307	1.0		0.999752
g000476	8000	0.002	0.999457	0.999766	1.0	0.999860

For input g001883, HSS and SBM had almost the same value of the cost function, while the solution configurations were truly different from each other. The result of DA for input g000644 is blank because DA can only manage 8192 variables.

**Table 8:** Results for large and medium classes, same as Table 1.

Input	Size	Density	HSS	SBM	DA	SA
g002312	6395	0.19	0.999957	0.999054	1.0	0.999930
g002563	6279	0.19	0.999842	0.999966	1.0	0.999945
g000495	5438	0.21	0.999941	0.999980	1.0	0.999958
g002204	5368	0.44	1.0	1.0	1.0	0.999903
g000503	5046	0.45	0.999954	0.999966	1.0	0.999983

**Table 9:** Results for large and dense classes, same as Table 1.

Input	Size	Density	HSS	SBM	DA	SA
g002527	5378	0.59	0.999949	0.999574	1.0	0.999885
g001345	5066	0.74	0.999252	0.999004	0.475147	1.0
p7000-2	7001	0.8	0.999992	0.999748	1.0	0.999563
g002300	5038	0.94	0.999970	0.999988	1.0	0.999995
g001651	5819	0.97	0.999949	0.999913	1.0	0.999930

**Table 10:** The lowest values of cost function found in this benchmarking for MQLib instances.

Input	Value of cost function	Solvers
g000989	− 2322	HSS, SBM, DA
g003215	− 821,734	HSS
g001269	− 45,661	HSS, SBM, DA
g000421	− 41,680.2	HSS, SBM
g002440	− 2,000,460	HSS, SBM, SA
g000432	− 188,363.1	HSS
g000524	− 4,335,188	HSS, SBM, DA, SA
g002586	− 7,161,694	HSS, SBM
g001327	− 9,267,492	HSS, SBM
g001469	− 1.42273e+07	HSS
g002600	− 41,194.45	HSS
g000969	− 6,647,406	HSS, SBM
g002898	− 1.276648e+07	HSS, SBM, DA
g001581	− 730,413.1	SBM
g000788	− 1,962,898	HSS
g000377	− 445,529	DA
g002569	− 5.084731e+08	HSS
g001086	− 3819.935	SA
g001337	− 4,634,430	DA
g000283	− 337,340.8	SA
g002512	− 327,679.6	DA
g000802	− 2,819,460	SBM
g003059	− 3,782,885	DA
g002332	− 4,586,683	SA
g002034	− 698,788.1	HSS
g003198	− 1.373565e+08	HSS
g002207	− 6,781,175	HSS, SBM, DA
g001913	− 1,177,002	HSS
g001393	− 358,732	SBM, DA
g002370	− 5.622634e+07	SA
imgseg-216041	− 9,572,357	HSS
imgseg-376020	− 1.376284e+07	HSS
g001883	− 403,013.1	SBM
g000644	− 132,820	SBM
g000476	− 106,794	DA
g002312	− 2.867864e+07	DA
g002563	− 5.848182e+07	DA
g000495	− 1.638467e+07	DA
g002204	− 1.229112e+08	HSS, SBM, DA
g000503	− 8.506962e+07	DA
g002527	− 8,261,389	DA
g001345	− 4.011876e+07	SA
p7000-2	− 1.824995e+07	DA
g002300	− 9.409027e+07	DA
g001651	− 130,005.8	DA

### NAE 3-SAT instances

**Figure 2:**
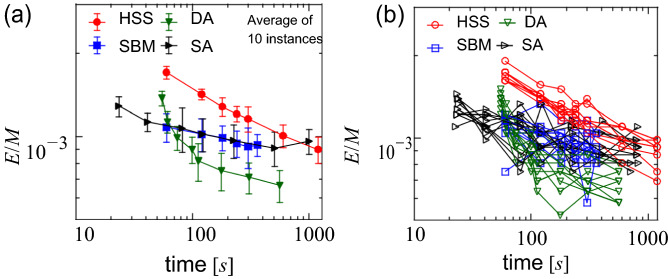
Value of the cost function per clause as a function of the execution time, obtained for NAE 3-SAT with a number of variables N = 8192 and a number of clauses M = 17,285, i.e., M/N ≈ 2.11. Each data point was obtained from an independent run. See the main text for the time metric of each solver. (**a**) Average of ten instances. The error bars denote standard deviation. For DA and SA, the execution time was also averaged. (**b**) Results for ten different instances.

Next, we present the results for the random NAE 3-SAT instances with a number of variables N = 8192 and a number of clauses M = 17285, i.e., instances with a clause-to-variable ratio N/M ≈ 2.11. Figure 2a shows the average of ten randomly generated instances of the cost function as a function of the execution time. As a reference, Fig. 2b shows the results for ten different instances. Given that each data point was obtained from an independent run, a longer run may lead to a worse solution than a shorter run. In the range 100–600 s, DA presented the lowest value of the cost function, closely followed by SBM and SA; HSS presented the highest value. In the region below 100 s, SBM and SA showed lower energy than DA. After a long-time calculation of about 1000 s, HSS finally reaches the same performance as SBM and SA, but still not as good as the result of 100 s run of DA. Interestingly, the performance of SBM and SA is almost identical for a wide range of execution time.

### SK model

**Figure 3:**
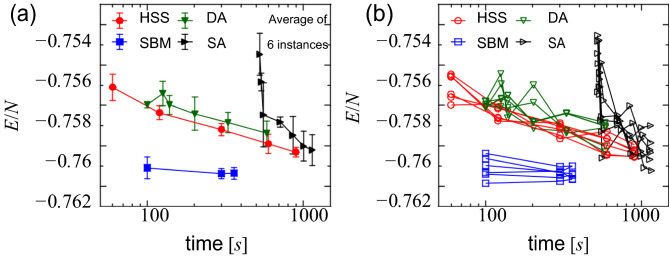
Value of cost function per variable as a function of the execution time, obtained for the SK model with a number of variables N = 8192 and J = 1. Each data point was obtained from an independent run. See the main text for the time metric of each solver. (**a**) Average of ten instances. The error bars denote standard deviation. For DA and SA, the execution time was also averaged. (**b**) Results for six different instances.

Finally, we present the results for the SK model with 8192 variables. As with the NAE 3-SAT instances, the experiments were performed by varying the execution time. Figure 3a shows the average of six randomly generated instances of the cost function as a function of the execution time. As a reference, Fig. 3b shows the results for six different instances. SBM clearly outperformed the other solvers, achieving the best solutions at the 100-s mark, with little energy change for longer runs. HSS and DA showed almost the same time dependence, although HSS provided a slightly better solution. It is interesting that this pair is different from the pair, SBM and SA, that exhibits similar performance in NAE 3-SAT instances. In runs longer than 600 s, SA obtained as good solutions as HSS and DA, but due to the all-to-all coupling, its pre-processing calculation was expensive, requiring at least approximately 500 s for the total calculation time.

## Discussion and conclusion

We benchmarked the heuristic QUBO solvers, HSS, SBM, DA, and SA, using the instances from the MQLib repository, random NAE 3-SAT, and the SK model. Benchmarking with problems of various origins revealed some of the characteristics of the strengths and weaknesses of each solver. For MQLib instances, which are a set of various problem instances including real-world problems, HSS showed the best performance on average, and SBM also showed stable performance that was not so different from HSS. DA outperformed other solvers on large instances, but it gave slightly poor solutions to some instances. It is rather natural result that the performance of DA varied depending on the instances because the performance of heuristic algorithms strongly depends on the problem instances in general, and it is somewhat surprising that HSS and SBM showed stable performance. In this experiment, with a run time of 5 min, we find that the difference in the value of cost function of the obtained solutions is often less than 0.01%, which is probably negligible in some application cases. Therefore, a possible direction for further study is to investigate how the results change in experiments with shorter run times. For random NAE 3-SAT instances at the SAT-UNSAT transition point, which is a typical hard optimization problem, DA performed best for most of the execution times. The performance of SBM and SA was almost the same, and HSS was the worst. It is believed that local search methods such as the parallel tempering method used in DA do not work well for SAT instances at the SAT-UNSAT transition point that have very few solutions^31^, and there is probably no efficient algorithm. Therefore, the result that DA still performed best implies that other solvers are also not particularly effective, which is as expected. For SK model, which is a typical hard problem originated from the spin glass, SBM exhibited a clear advantage over other solvers, while HSS and DA showed similar performance. Since the parallel tempering method is considered to work relatively well for the SK model, it is a bit surprising that SBM, rather than DA, showed outstanding performance as opposed to the case of NAE 3-SAT. It is an important challenge to understand the characteristics of each solver found in this study from the viewpoint of their algorithm and hardware architecture.

## Data Availability

All other data used in this study are available from the corresponding authors upon reasonable request. The problem instaces of MQlib is available from the MQLib repository^36^. The NAE 3-SAT and SK model instance was generated reproduced by the python program shown in Listings 1 and 2 with Python 3.8.2 on Ubuntu20.04.3 LTS.

**Figure:**
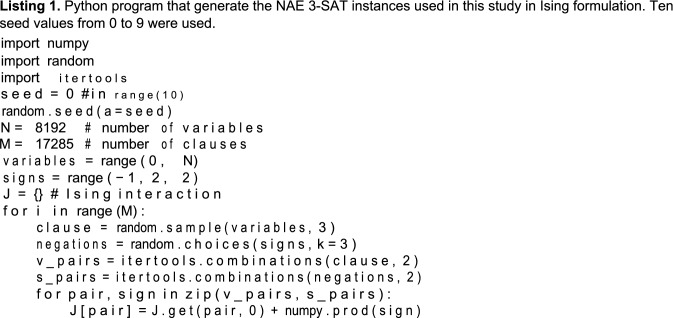

**Figure:**